# Diffuse large B-cell lymphoma presenting with central pontine myelinolysis: a case report

**DOI:** 10.1186/s13256-015-0614-8

**Published:** 2015-06-05

**Authors:** Eri Kawata, Reiko Isa, Junko Yamaguchi, Kazuna Tanba, Yasuhiko Tsutsumi, Yoshinari Nagakane, Hitoji Uchiyama, Teruaki Akaogi, Yutaka Kobayashi, Nobuhiko Uoshima

**Affiliations:** 1 Department of Hematology, Japanese Red Cross Kyoto Daini Hospital, 355-5 Haruobi-cho, Kamigyo-ku, Kyoto 602-8026 Japan; 2 Department of Hematology and Oncology, Kyoto Prefectural University of Medicine, 465 Kajii-cho, Kamigyo-ku, Kyoto 602-8566 Japan; 3 Department of Neurology, Japanese Red Cross Kyoto Daini Hospital, 355-5 Haruobi-cho, Kamigyo-ku, Kyoto 602-8026 Japan; 4 Department of Hematology, Japanese Red Cross Kyoto Daiichi Hospital, 15-749 Honmachi, Higashiyama-ku, Kyoto 605-0981 Japan

**Keywords:** Central pontine myelinolysis, Diffuse large B-cell lymphoma, Hyponatremia

## Abstract

**Introduction:**

The most common cause of central pontine myelinolysis is an overly rapid correction of hyponatremia, although it can also occur in patients with any condition leading to nutritional or electrolyte stress. We report a case of diffuse large B-cell lymphoma with central pontine myelinolysis developing at the onset of disease. To the best of our knowledge, hematological malignancies presenting with central pontine myelinolysis have been rarely reported, especially in previously untreated patients, as in our case report.

**Case presentation:**

A 78-year-old Japanese woman presented to a neighborhood clinic with persistent high fever, edema, and general weakness. Despite the absence of specific neurological findings, brain magnetic resonance imaging showed an abnormal lesion in the central pons area of her brain (hyperintense on T2-weighted and hypointense on T1-weighted sequences), compatible with central pontine myelinolysis. She was admitted to our emergency department in a state of shock one month later. The results of her blood tests showed greatly elevated C-reactive protein and lactate dehydrogenase levels. She had severe hypoalbuminemia and mild hyponatremia, and showed signs of disseminated intravascular coagulation. Mild bilateral pleural effusion, prominent subcutaneous edema, and splenomegaly were detected on her systemic computed tomography scan. Her body fluid cultures did not show signs of infection and her spinal aspiration did not show pleocytosis or abnormal cells. A diagnosis of diffuse large B-cell lymphoma was made based on the results of her bone marrow examination. As she was critically ill before the diagnosis was made, she was treated with methylprednisolone pulse therapy, followed by systemic chemotherapy (rituximab with modified THP-COP regimen, including cyclophosphamide, pirarubicin, vindesine, and prednisolone), which resulted in complete remission and recovery without any neurological defects, and resolution of her abnormal findings on magnetic resonance imaging.

**Conclusions:**

Central pontine myelinolysis is a serious condition that may result in neuropathological sequelae and mortality, and clinicians should be aware of its potential presence in patients with malignancies.

## Introduction

Central pontine myelinolysis (CPM) is a neurological disorder caused by damage to the myelin sheath of nerve cells in the brainstem. It is a clinically heterogeneous disease, originally thought to occur only in the central pons area [1, 2], and later shown to affect areas outside the pons [3]. When demyelination involves areas outside the pons, the disorder is referred to as either extrapontine myelinolysis (EPM) or central and extrapontine myelinolysis (CPEPM). The most common cause of these disorders is an overly rapid correction of hyponatremia in patients with conditions associated with nutritional or electrolyte stress. CPEPM is sometimes associated with poor prognosis and may cause permanent cognitive impairment. Here, we describe the case of a patient with malignant lymphoma presenting with CPM at the onset of the disease, who showed complete recovery without any sequelae.

## Case presentation

A previously healthy 78-year-old Japanese woman with mild diabetes mellitus and hypertension presented to a neighborhood clinic with general fatigue since July, and progressive muscle weakness and walk disturbances since September 2012. She had no specific family medical history. She complained of progressive illness and fever in November 2012, and was diagnosed with severe edema of the lower extremities; however, no specific neurological abnormalities were detected except mild muscle weakness in the lower extremities. Her blood tests at that time showed a normal white blood cell (WBC) count (5.2 × 10^9^/L), mild hyponatremia (sodium level 133mEq/mL), and elevated levels of C-reactive protein (CRP) (16.23mg/dL) and lactate dehydrogenase (LDH) (950U/L). No abnormal findings were detected on her ultrasound tomography or systemic computed tomography (CT) scans. Magnetic resonance imaging (MRI) of her brain revealed a hyperintense lesion in the central pons area in T2-weighted imaging, diffusion-weighted imaging (DWI), and fluid-attenuated inversion recovery (FLAIR) imaging with no mass effect.

**Fig. 1:**
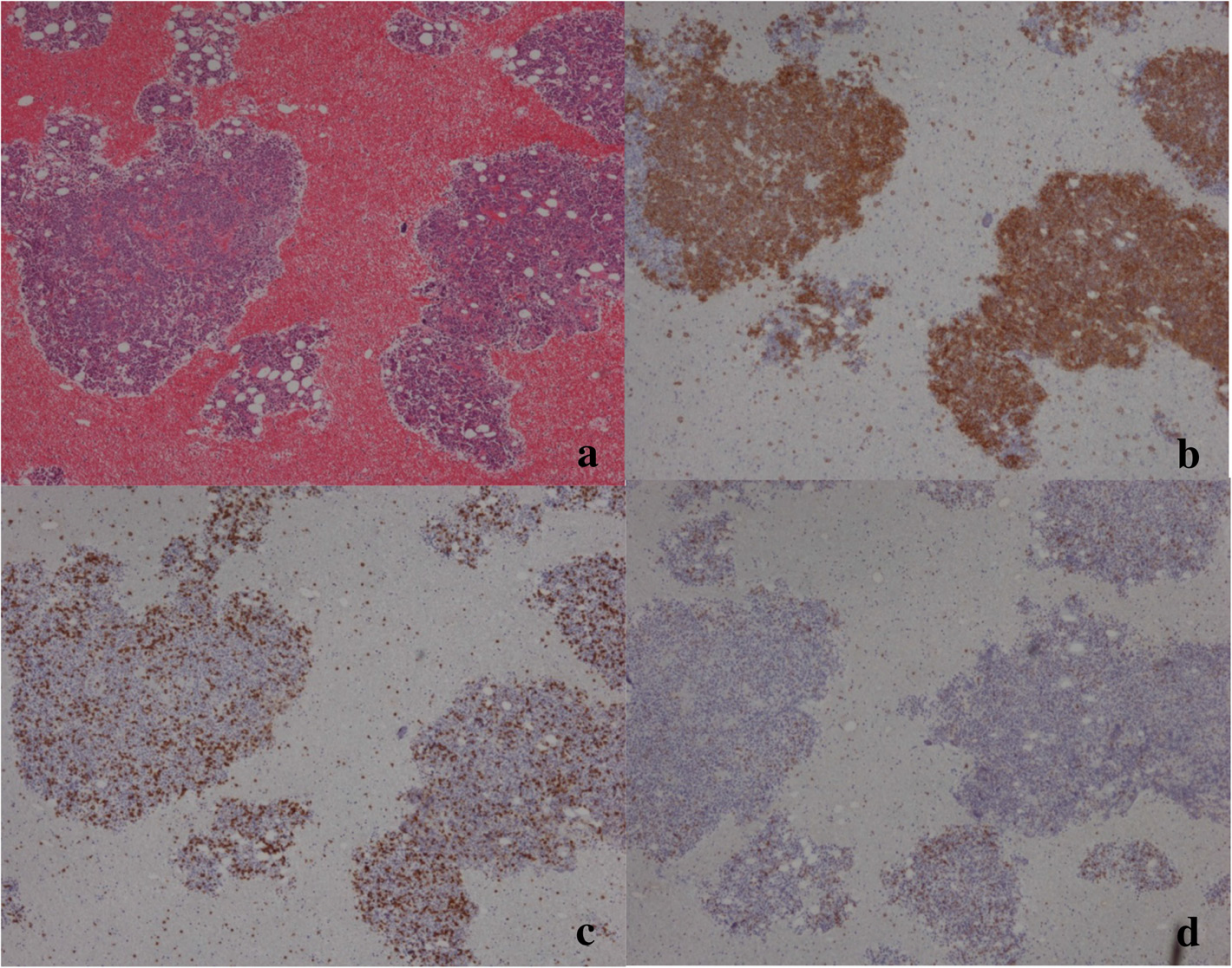
Bone marrow specimen. Bone marrow aspiration revealed a hypercellular bone marrow with numerous large lymphoma cells. (**a**) Hematoxylin and eosin staining (40x magnification) was (**b**) positive for CD20 and (**c**) MT1, and (**d**) negative for CD3

**Fig. 2:**
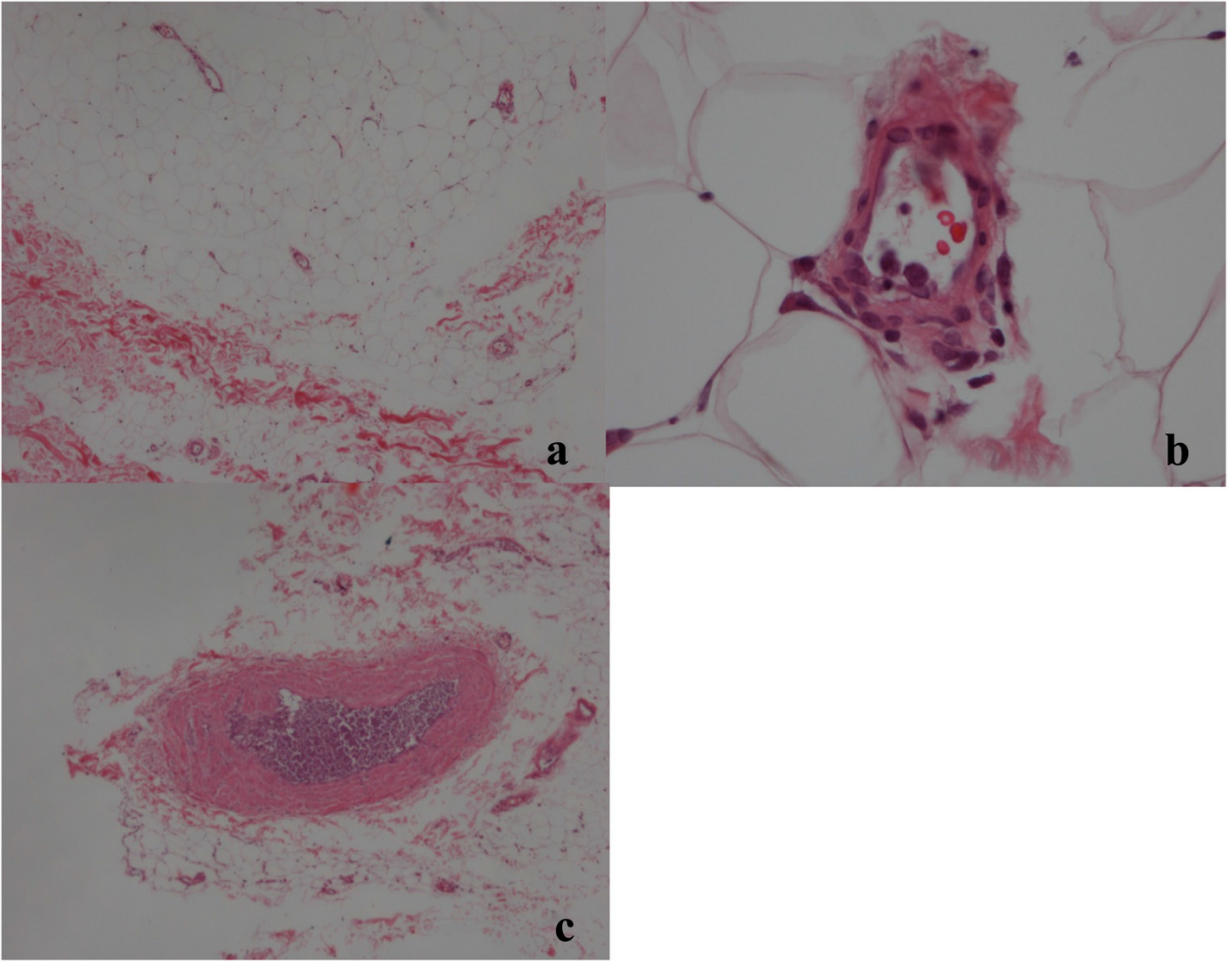
Skin specimen. Large lymphoma cells were also found in the vessels of the epidermis and dermis. Cells were observed in microvessels (hematoxylin and eosin staining: (**a**) 40x, (**b**) 400X) but were more prominent in larger vessels ((**c**) 40X)

She was admitted to our emergency department in a state of shock in December 2012. Her blood tests showed greatly elevated CRP (25.25mg/dL) and LDH (1475U/L) levels, an almost normal WBC count (9.4 × 10^9^/L), and a low platelet count (94 × 10^9^/L), which decreased to 48 × 10^9^/L and 40 × 10^9^/L, respectively, in the following days. She had severe hypoalbuminemia (1.64g/dL) and mild hyponatremia (sodium 132mEq/mL), and showed signs of disseminated intravascular coagulation. A lumbar puncture showed that her cerebrospinal fluid pressure was within normal limits, with no pleocytosis, abnormal cells, or elevation of sugar or protein. Mild bilateral pleural effusion, prominent subcutaneous edema, and splenomegaly were detected on her systemic CT scan. Her body fluid cultures (blood, urine, sputum, and cerebrospinal fluid) did not show signs of infection, and she tested negative for human immunodeficiency virus infection. Her bone marrow examination showed a hypercellular bone marrow with numerous large lymphoma cells. A G-band analysis revealed complex karyotypes, and immunoglobulin heavy chain (IgH)-J_H_ gene rearrangement was detected. Lymphoma cells, which were positive for CD20 and MT1 and negative for CD3 (Fig. 1), were also present in vessels, particularly in larger vessels, but not in capillary vessels, in the epidermis, or dermis (Fig. 2).

**Fig. 3:**
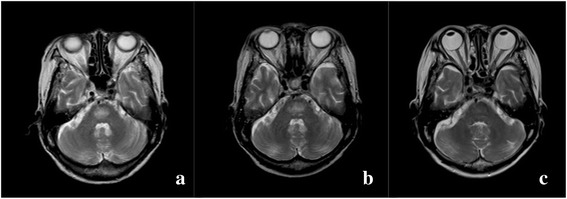
Brain magnetic resonance scan. The hyperintense lesion in the central pons in T2-weighted magnetic resonance imaging decreased and was almost undetectable after several courses of chemotherapy. (**a**) Before treatment, (**b**) after two courses, and (**c**) after seven courses of chemotherapy

She was diagnosed with stage IVB diffuse large B-cell lymphoma (DLBCL) and identified as high risk according to the international prognostic index score. As she was critically ill before the diagnosis was made (she required mechanical ventilation and central venous catheter insertion), she was treated with methylprednisolone pulse therapy, followed by systemic chemotherapy (rituximab with modified THP-COP regimen, including cyclophosphamide, pirarubicin, vindesine, and prednisolone). Despite the presence of grade 4 neutropenia, her physical condition improved markedly after her first course of chemotherapy, with gradual correction of her hyponatremia and hypoalbuminemia. After two courses of chemotherapy, no lymphoma cells were detected on her bone marrow examination, and the hyperintense lesion in the central pons on T2/FLAIR imaging had decreased in size and intensity. After four courses of chemotherapy, the hyperintense lesion was even smaller, with no enhancement on gadolinium imaging, and after seven courses, it was almost undetectable (Fig. 3). Her serum sodium levels showed a slow and gradual correction: 132mEq/mL at presentation to our department, 137mEq/mL after two courses of chemotherapy, and 140mEq/mL after seven courses of chemotherapy. A complete response was achieved and sustained after nine courses of chemotherapy. She showed complete recovery and was discharged without any sequelae. However, she relapsed nine months after her last chemotherapy. She was retreated with salvage chemotherapy and showed a partial response, but eventually died of lymphoma five months later.

## Discussion

CPM is a neurological disease caused by damage to the myelin sheath of nerve cells in the brainstem. It was first described in three patients with a history of alcohol abuse and one patient with malnutrition by Adam *et al*. in 1959 [2]. The defining neuropathological characteristics of CPM are systemic, sharply demarcated lesions, with selective destruction of myelin sheaths and an absence of inflammatory infiltrates. Blood vessels are patent, and both axons and neuronal cell bodies are generally spared. Since both the pontine and extrapontine sites involved have a rich grey and white matter interface, it has been hypothesized that rapid osmotic changes result in endothelial injury in the more vascular grey and white matter, inducing the release of myelinolytic factors that damage the adjacent white matter [1]. MRI is more accurate than CT for the detection of CPEPM; lesions are identified as hyperintense on T2-weighted (including FLAIR) and hypointense on T1-weighted MRI sequences with no mass effect. Early DWI changes are another common finding [4, 5]. The most common cause of CPEPM is an overly rapid correction of hyponatremia in patients with conditions leading to nutritional or electrolyte stress, such as alcoholism, liver disease, immunosuppression, malnourishment, gastrointestinal diseases with acute electrolyte abnormalities, syndrome of inappropriate antidiuretic hormone secretion, renal disease, cancer, pregnancy, and high-endurance exercise [3, 6, 7].

Although the exact pathophysiology of CPEPM is not completely understood, a rapid increase in serum tonicity in patients with severe hyponatremia who have made intracellular adaptations to hypotonicity is widely recognized as the underlying problem. The clinical presentation varies among patients, with some showing no obvious neurological symptoms and only abnormal MRI findings, and others showing a combination of neuropsychiatric (such as emotional lability, disinhibition, and other bizarre behaviors) and neurologic (such as confusion, impaired cognition, dysarthria, dysphasia, gait instability, weakness or paralysis, and generalized seizures) symptoms [1, 8, 9]. The extent of radiologic signal abnormality or the severity of hyponatremia may not be associated with clinical outcomes. Serial brain imaging is important in suspicious cases because a substantial proportion of patients have normal findings on initial MRI scans [10]. The outcome of patients with CPEPM is traditionally reported as poor; however, several studies have shown that this is not inevitable. A retrospective study conducted by Graff-Radford *et al*. reported a favorable outcome in 60% of patients, and an overall mortality of 8% in the acute setting [10]. Similarly, in a large series of 34 patients with CPM, 65% of patients achieved a good or moderate outcome (no functional deficit or independence despite minor deficits), and 27% showed a poor outcome, including a 6% mortality rate [11]. The increasing availability of MRI, which facilitates early diagnosis, and improvements in intensive care treatments are likely contributing factors to the noted improvements in outcomes.

## Conclusions

Our case report describes a case of a woman who presented with an abnormal lesion in the central pons area (hyperintense in T2-weighted, DWI, and FLAIR imaging, and hypointense in T1-weighted imaging) compatible with CPM at the first onset of DLBCL. Her serum sodium level was stable before and after the onset of CPM. Lymphoma cells were not detected in her cerebrospinal fluid, and her brain MRI showed neither mass effect nor gadolinium-enhanced lesions. In the absence of pathological brain specimens at the time of diagnosis, other diagnostic possibilities should be considered. However, in our patient, the lack of abnormal cerebrospinal fluid findings, the absence of enhancement, and the extremely localized lesion made the diagnosis of intracranial involvement of DLBCL difficult.

To the best of our knowledge, few reports have described cases of hematological malignancies presenting with CPM, especially in previously untreated patients, such as the present case [12–14]. In these reports, pathological specimens were inaccessible, and the diagnosis of CPM rested on imaging studies alone, similar to our case. Although the cause of CPM was not fully determined, the underlying chronic mild hyponatremia and severe hypoalbuminemia caused by the lymphoma were possible triggers of CPM in our patient. She achieved complete response of her lymphoma, and no neurological symptoms or sequelae were observed after several courses of chemotherapy. However, CPEPM is a severe and sometimes preventable condition that can result in serious neuropathological sequelae and even death. As the etiology of CPEPM remains unclear and effective treatment strategies have not been developed, clinicians should be aware of this condition in patients with malignancies, even at first diagnosis, because nutritional and electrolyte stress may increase the vulnerability of these patients.

## Consent

Written informed consent was obtained from the patient’s son for publication of this case report and accompanying images, as the patient had died at the time of submission of the article. A copy of the written consent is available for review by the Editor-in-Chief of this journal.

## Abbreviations

CPM: central pontine myelinolysis
CPEPM: central and extrapontine myelinolysis
CRP: C-reactive protein
CT: computed tomography
DLBCL: diffuse large B-cell lymphoma
DWI: diffusion-weighted imaging
EPM: extrapontine myelinolysis
FLAIR: fluid-attenuated inversion recovery
IgH: immunoglobulin heavy chain
LDH: lactate dehydrogenase
MRI: magnetic resonance imaging
WBC: white blood cell
